# Case Report: Possible association between dermatomyositis and Trastuzumab Deruxtecan therapy of triple-negative breast cancer patient

**DOI:** 10.3389/fimmu.2025.1636581

**Published:** 2025-11-10

**Authors:** Huan Han, Maksim Semenov, Chao Li, Yuhong Zhang, Taoyan Lin, Ping Zheng, Jing Cai

**Affiliations:** 1 Nanfang Hospital of Southern Medical University, Department of Pharmacy, Guangzhou, Guangdong, China; 2 Shaanxi Province People’s Hospital, Pharmaceutical Department, Xi’an, Shaanxi, China; 3 Cancer Hospital of the Chinese Academy of Medical Sciences, Department of Pharmacy and Clinical Trials, Shenzhen, Guangdong, China; 4 Sun Yat-sen University Cancer Center, Department of Clinical Pharmacy, Guangzhou, Guangdong, China

**Keywords:** breast cancer, Trastuzumab Deruxtecan (T-DXd), dermatomyositis, interstitial pneumonia, adverse drug reaction, antibody-drug conjugate

## Abstract

**Introduction:**

we present the case of the first documented occurrence of concurrent dermatomyositis and interstitial pneumonia associated with Trastuzumab Deruxtecan (T-DXd) therapy. We hypothesize that T-DXd likely induced an autoimmune response through tumor antigen release, resulting in multi-system involvement of skin, muscle, and pulmonary tissues. The shared pathogenesis of dermatomyositis and interstitial pneumonia involves aberrant activation of the type I interferon pathway, NF-κB signaling, and TGF-β cascade, collectively driving inflammatory and fibrotic processes. Notably, progression of breast cancer temporally coincided with the onset of dermatomyositis. Although anti-TIF1-γ antibodies were not detected, the possibility of paraneoplastic dermatomyositis cannot be definitively excluded.

**Patient’s concerns and clinical findings:**

a 43-year-old woman initially diagnosed with Luminal B-type invasive ductal carcinoma of the left breast and experienced tumor recurrence after modified radical mastectomy. Lymph node biopsy confirmed triple-negative breast cancer (cT2N3cM0, stage IIIC) with HER2 ultra-low expression (1+). During palliative second-line treatment with Trastuzumab Deruxtecan, patient developed typical dermatomyositis symptoms and proximal muscle weakness 7 days after the first cycle of administration. Following glucocorticoid treatment, dermatomyositis symptoms showed partial relief. However, with continued administration of T-DXd in compliance with original protocol (cycles 2-6), the manifestation of dermatomyositis significantly worsened. The condition remained poorly controlled despite administration of Prednisone combined with Hydroxychloroquine, and showed no significant improvement after adding methotrexate. Concurrent tumor evaluation revealed disease progression, too. Following the third treatment cycle, chest CT revealed interstitial pneumonia.

**Conclusion:**

reported here findings suggest that T-DXd may trigger multi-system involvement through immune-mediated mechanisms, resulting in drug-induced dermatomyositis and interstitial pneumonia. Our findings highlight the need for heightened vigilance regarding such immune-related adverse events during T-DXd therapy, where early recognition and intervention are critically important to avoid irreversible and possibly fatal complications.

## Introduction

Breast cancer remains one of the most prevalent malignancies among women worldwide, where the expression level of human epidermal growth factor receptor 2 (HER2) plays a pivotal role in both therapeutic decision-making and prognostic stratification. Patients with HER2-positive breast cancer benefited significantly from HER2-targeted therapies, while HER2-negative tumors remained relegated to conventional chemotherapy until nowadays. However, recent studies have revealed that HER2-low breast cancer defined as tumors with immunohistochemistry (IHC) scores of 1+ or 2+ without gene amplification accounts for a substantial proportion (approximately 60%) of all breast cancer cases. Previously classified as HER2-negative, these patients were ineligible for HER2-targeted therapies (1, 2). Trastuzumab deruxtecan (T-DXd) is a novel antibody-drug conjugate (ADC) composed of three key components: humanized anti-HER2 IgG1 monoclonal antibody (Trastuzumab) for tumor-selective targeted delivery, cleavable tetrapeptide-based linker and potent topoisomerase I inhibitor payload (Deruxtecan) (3). Collectively, this enables highly efficient delivery of cytotoxic payloads to HER2-low expression tumor cells, with subsequent release in the tumor microenvironment (TME), thereby exerting potent antitumor activity. DESTINY-Breast04 trial (NCT03734029) demonstrated statistically significant and clinically meaningful efficacy of T-DXd in patients with HER2-low metastatic breast cancer (4).

Dermatomyositis (DM) is an autoimmune disorder characterized by chronic inflammatory damage of both skeletal muscles and skin, with an estimated incidence of 1 per 100,000. While most cases are idiopathic, 15-30% of adult-onset dermatomyositis represented as a paraneoplastic syndrome (5, 6). However, rarely but dermatomyositis has been documented as a potential immune-related adverse event (irAE) associated with certain classes of anticancer agents (7–10).

## Case presentation

**Figure 1:**
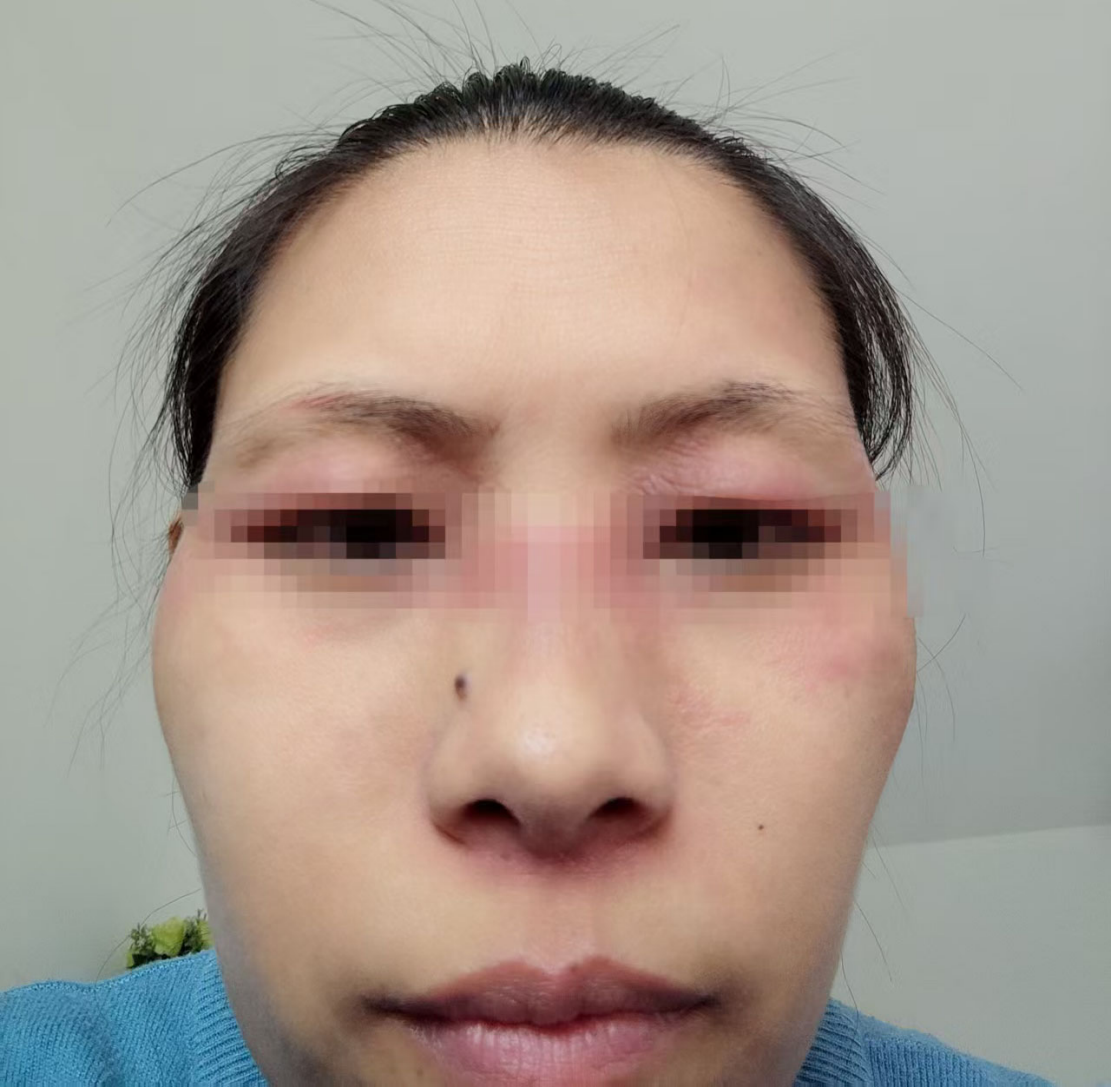
Dermatomyositis onset after the first cycle of Trastuzumab Deruxtecan treatment.

In December 2022, a 43-year-old Chinese female with neither comorbidities nor personal or familial history of autoimmune diseases was diagnosed with left breast invasive ductal carcinoma. Immunohistochemistry confirmed Estrogen Receptor (ER) weakly positive (20%), Progesterone Receptor (PR)-negative, Androgen Receptor (AR) weakly positive (10%), Her-2 (0), mutant P53-positive (90%), Ki-67 positive (80%) tumor. Under general anesthesia patient underwent a modified radical mastectomy of the left breast. Intraoperative frozen section examination revealed metastatic carcinoma in the sentinel lymph node (approximately 3 mm in diameter), with no evidence of metastasis in the left axillary lymph nodes. Starting from January 2023, patient received 4 courses of adjuvant AC-T chemotherapy (Epirubicin + Cyclophosphamide followed by Paclitaxel Liposome). Post-chemotherapy treatment included regular endocrine therapy with Tamoxifen and Goserelin, as well as radiotherapy with exposure doses PTV-CW 48Gy/25F, PTV-SC 48Gy/25F. In March 2024, after disease progression, patient was diagnosed with multiple lymph node metastases (cT2N3cM0, Stage IIIC). After biopsy of the left supraclavicular lymph node with follow up IHC, tumor was characterized as ER-negative, PR-negative, Her-2 (0:90%;1+: 8%; 2+: 2%; total grade: 1+), AR-negative, mutant P53-positive (80%), Ki-67-positive, (90%), TROP-2 (3+), PD-L1 (CPS≈30, clone SP263). Starting from April 2024, patient received Gemcitabine and Carboplatin with further disease progression onset after 6 cycles of treatment. November 2024, therapy with Trastuzumab Deruxtecan was thereafter initiated. Seven days post-infusion, inching erythematous papules appeared on the lateral side of both thighs, rapidly progressing over the whole body (**Figure 1**). Orally administered Loratadine was ineffective, so treatment was switched to oral Prednisone 15 mg twice daily, which stabilized the condition. After 3 days, the dose was reduced to 15 mg once daily. Skin rash was alleviated. However, new lesions continued to appear and patient was referred to dermatology department. Observation revealed violaceous edematous patches centered on both upper eyelids and flat violaceous papules symmetrically distributed on the extensor surfaces of the metacarpophalangeal joints, poikilodermatous on the neck and chest, typical Heliotrope syndrome, Gottron syndrome, V-syndrome. At the same time, patient had decreased limb muscle strength, symmetrical proximal muscle weakness and myalgia.

## Diagnostic assessment and therapeutic intervention

**Figure 2:**
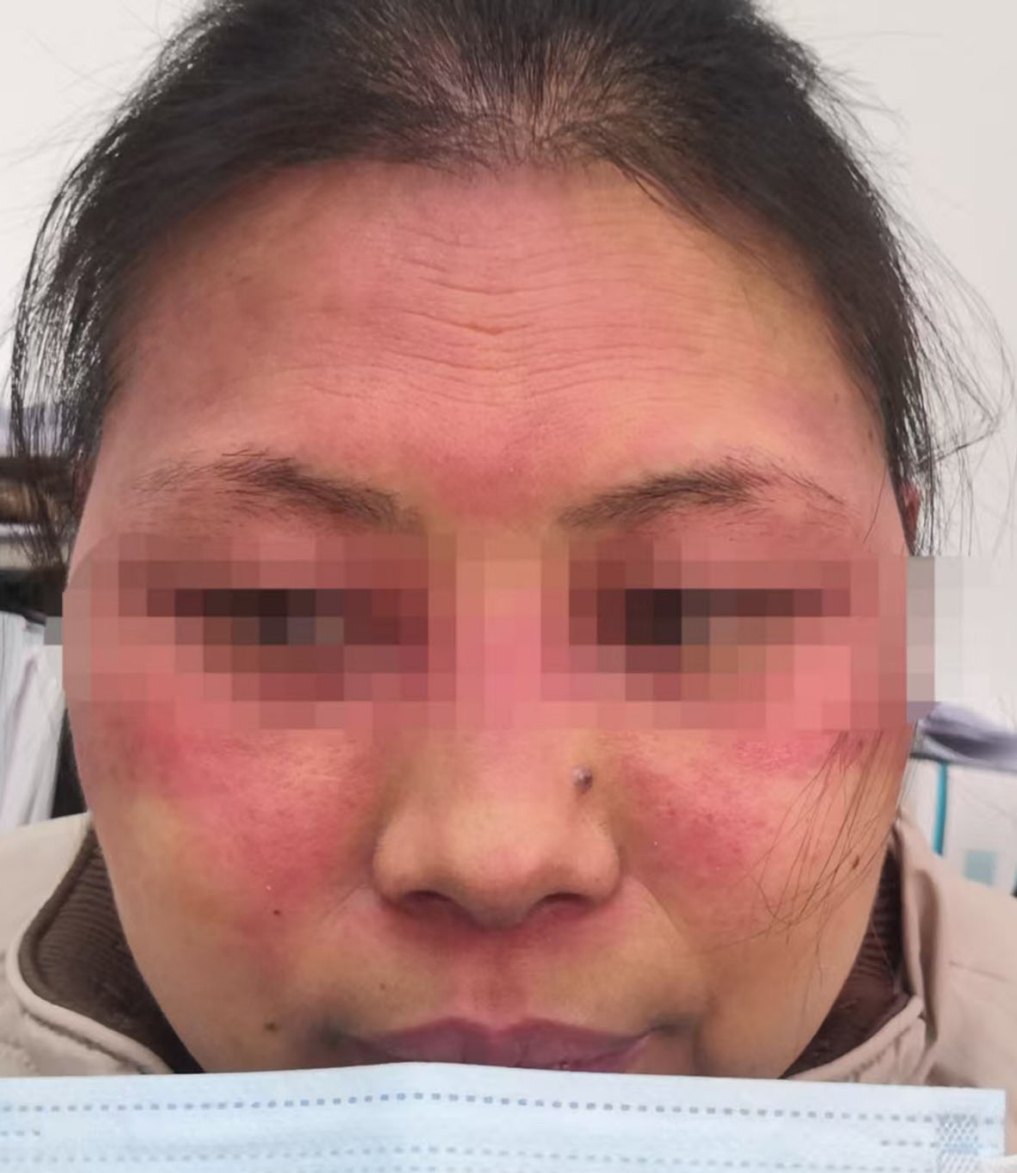
Recurrence and worsening of dermatomyositis symptoms after the third cycle of Trastuzumab Deruxtecan administration.

**Figure 3:**
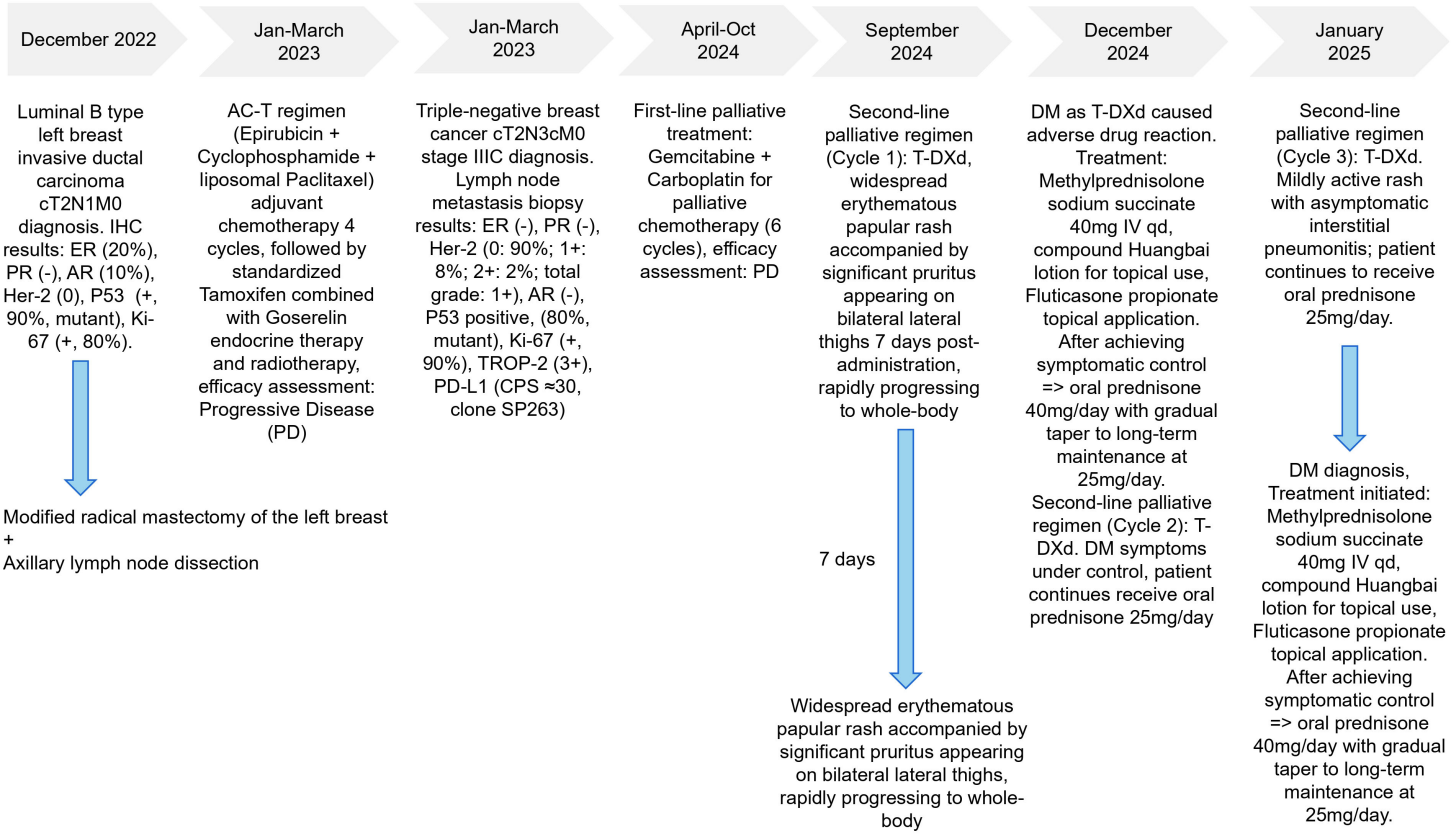
Treatment history of this case.

Laboratory investigation results displayed α-Hydroxybutyrate Dehydrogenase (HBDH) 247U/L (range 90–180 U/L), Creatine Kinase (CK) 511U/L (range 40–200 U/L), C-reactive protein (CRP) 4.59mg/L(range 0.00-6.00 mg/L), Erythrocyte Sedimentation Rate (ESR) 25mm/1h (range 0–20 mm/1h), Ro52-positive (+++), Antinuclear Antibody (ANA) weakly positive (±), other Myositis-Specific Antibodies Jo-1, ssB, Sm, UI-nRNP, SCL-70, ARPA/Rib-P, Proliferating Cell Nuclear Antigen (PCNA), Mitochonfria antibody AMAM2, Anti-Centromere Antibody CENPB, PM-Scl, ssA, Anti-Nucleosome Antibody AnuA, anti-histone antibodies (AHA) all were negative, while Mi-2, TIF-1, MDA5 levels were not examined. HCV and HIV tests were negative; blood glucose, electrolytes, HbA1c, liver/kidney function, pro-BNP, hepatitis B, and procalcitonin results showed no abnormalities. Electromyography (EMG) demonstrated myopathic changes. Skin biopsy revealed focal keratinocyte necrosis, normal epidermal thickness with vacuolar interface changes, dermal superficial vascular dilation, perivascular lymphocytic infiltration. Finally, based on these results, patient was diagnosed with drug-induced dermatomyositis. Eczema and atopic dermatitis were excluded. Trastuzumab Deruxtecan was suspected likely to be the causative agent of the adverse reaction, while patient medical history had no evidence of genetic predisposition, pre-existing autoimmune diseases, viral infections, and UV exposure. Patient was treated with IV Methylprednisolone sodium succinate once daily 40mg/day (after disease progress control was achieved, patient was given oral Prednizolone 40 mg/day instead and further dose was reduced to 25 mg/day for long-term treatment) and compound Huangbai lotion + topical Fluticasone Propionate. Considering the patient’s poor response to previous triple-negative breast cancer treatment attempts and limited availability of therapeutic options, as well as the fact that the dermatomyositis symptoms were improved after steroid therapy administration, T-DXd treatment was not discontinued. Notably, after administration of the same dose of T-DXd during the second cycle, recurrence of dermatomyositis symptoms was not observed. However, after the third cycle of Trastuzumab Deruxtecan administration, patient experienced mild reactivation of dermatomyositis (**Figure 2**). Additionally, chest CT revealed interstitial pneumonia. January 2025, following the fourth cycle of Trastuzumab Deruxtecan administration, patient’s dermatomyositis symptoms severely aggravated. Thus, oral Methotrexate 5 mg once per week was added to the treatment regimen (the entire treatment history is illustrated in **Figure 3**). Further antitumor treatment response evaluation indicated disease progression.

## Discussion

Dermatomyositis (DM) is a chronic inflammatory disorder affecting both skin and muscles. Pathogenesis involves complexity of specific factors such as inheritance, immune system dysregulation, environmental influence and aberrant molecular signaling pathways. Genetic factors play a significant role in the pathogenesis of dermatomyositis, alleles HLA-DRB1*03:01 and HLA-DQA1*05:01 are strongly associated with disease susceptibility (11). Immune system disregulations play key role in pathogenesis of dermatomyositis. For instance, Anti-MDA5 antibodies are associated with skin lesions and interstitial lung disease, while anti-TIF1-γ antibodies are linked to cancer-associated dermatomyositis, anti-Mi-2 antibodies, in turn, correlate with immune disorders driven skin diseases (12, 13). Above mentioned antibodies triggers muscle and skin local inflammatory responses through activation of B cells, T cells, dendritic cells and complement system. Dermatomyositis has traditionally been considered a humoral-mediated disease (14, 15). However, recent findings increasingly support alternative mechanisms of dermatomyositis pathogenesis, for example, cell-mediated immunity defects and innate immune system dysfunction. Pathological changes in dermatomyositis-affected muscles include infarctions, perifascicular atrophy, endothelial cell swelling and necrosis, membrane attack complex (MAC) deposition in vessel walls, and elevated levels of MHC-I in myocytes (16). More evidences suggested that T cell-mediated cytotoxicity may contribute to muscle inflammation and damage during dermatomyositis pathogenesis. Bank et al. demonstrated that autologous peripheral blood CD3+ cell clones isolated from dermatomyositis patients induce cell-mediated myocytotoxicity *in vitro*, which can be blocked by OKT3 (an anti-CD3 monoclonal antibody) (17). Furthermore, identified from dermatomyositis patient tissue muscle-infiltrating CD8+ T-cell specifically vectorial oriented perforin supported the evidence of T cell-mediated cytotoxicity (18). Taking into account, that CD4+ plasmacytoid dendritic cells (pDCs) can activate the adaptive immune system. It is possible that multiple immune pathways are involved in DM pathogenesis (19, 20). Regarding pathological changes in skin, histopathological findings included hyperkeratosis, vacuolar degeneration and apoptosis of epidermal basal cells, increased dermal mucin deposition, and cell-poor interface dermatitis. At the molecular signaling level, activation of the type I interferon pathway, NF-κB, and mTOR pathways plays a pivotal role in driving inflammatory responses and tissue damage. Additionally, paraneoplastic dermatomyositis (PDM) recently attracted significant attention, as approximately 15-30% of DM patients have associated malignancies, with the highest risk of occurrence within 3 years before or after DM was diagnosed. The pathogenesis remains poorly understood, but several studies suggested molecular mimicry between tumor associated antigens and self-antigens as a primary mechanism. Thus, tumor-released antigens may trigger cross-reactive autoantibody production. A retrospective cohort study demonstrated associations between anti-transcription intermediary factor 1 (TIF1)-γ and anti-nuclear matrix protein 2 (NXP2) antibodies with stage III+ cancer-associated myositis (CAM) (21). Such relevance is particularly observed in patients with anti-TIF1-γ antibodies. Environmental factors such as viral infections (e.g., HIV, HCV) may also contribute to DM pathogenesis (22), as well as UV radiation (23). Additionally, drug-induced dermatomyositis frequent occurrence was most commonly associated with medications such as Hydroxyurea, immune checkpoint inhibitors, statins, Penicillamine, and TNF inhibitors. Time from drug first administration to DM symptoms manifestation ranges from 21 to 288 days. Some drug-induced DM patients histopathology analysis revealed vacuolar interface dermatitis with mucin deposition. Those patients were also positive for antinuclear antibodies and anti-TIF1-γ, anti-Jo-1, anti-Mi-2, anti-Ro antibodies 30.9%, 6.7%, 3.0%, 2.4% and 2.4%, respectively. Serum aldolase, creatine kinase, lactate dehydrogenase, CRP levels and erythrocyte sedimentation rate were elevated for 18.8%, 37.6%, 13.2%, 12.7% and 10.3% respectively (7).

We hypothesized that the DM symptoms were triggered by T-DXd. As mentioned, patient had no prior history of autoimmune diseases, skin or muscle effecting disorders or remarkable family history. December 2022, diagnosed with left breast invasive ductal carcinoma, 2 years later our patient developed DM symptoms 7 days after initiating T-DXd therapy. While symptoms initially ameliorated due to the glucocorticoid therapy, later, after three additional treatment cycles, DM got worsened. In addition, CT revealed interstitial pneumonia. Collectively, it raised suspicions about drug adverse reaction (ADE). We explained absence of DM exacerbation by T-DXd after the second cycle of its administration as an effect of corticosteroid therapy. However, later aggravation of DM symptoms may reflect cumulative dosing effects of T-DXd, since 25 mg prednisone could no longer maintain immune equilibrium. Feb 2024 PubMed and FAERS database search revealed no prior reports of dermatomyositis as an adverse reaction to Trastuzumab Deruxtecan administration. The DESTINY-Breast04 trial results reported that drug-related interstitial lung disease or pneumonitis occurred in 12.1% of the patients who received Trastuzumab Deruxtecan (4). Received T-DXd therapy, our patient was presented not only with classic dermatomyositis manifestations but also with mild interstitial pneumonia confirmed by the chest CT. Some studies has also mentioned that DM may be complicated by interstitial pneumonia in patients often positive for ANA and anti-Jo-1 antibodies, along with elevated ESR (24). Thus, in our case, positive antibodies for ANA and anti-Ro52 and elevated ESR supports diagnosis of dermatomyositis complicated by interstitial pneumonia. Dermatomyositis and interstitial pneumonia share common pathogenic mechanisms, for example, activated IFN-α/β signaling pathway driving immune cell infiltration and inflammation. In turn, elevated levels of IL-6, TNF-α, and IL-1β result in inflammation aggravation and tissue damage. Activation of the TGF-β signaling pathway promotes fibrosis in both muscle and skin tissues in DM patients, while in interstitial lung disease patients, TGF-β drives pulmonary fibroblast activation, resulting in collagen deposition and pulmonary fibrosis. In both dermatomyositis and interstitial lung disease, reactive oxygen species (ROS) and reactive nitrogen species (RNS) induce cellular damage and inflammation by damaging DNA, proteins, and lipids, causing cellular damage and inflammation. Microvascular endothelial injury is an important hallmark of DM, while in interstitial lung disease, pulmonary microvascular endothelial damage may lead to disruption of the alveolar-capillary barrier, therefore, aggravating inflammation and fibrosis (25). Thus, we hypothesized that T-DXd might be triggering immune dysregulation as an adverse drug reaction, effected the lungs, skin, and muscles, thereby inducing interstitial pneumonia and dermatomyositis. However, rare case reports describe Trastuzumab (non-ADC formulation) drug-induced DM as an adverse reaction. One of such cases introduced invasive ductal breast carcinoma patient developed dermatomyositis after six cycles of Trastuzumab therapy, that was confirmed as a drug-induced adverse reaction. Authors concluded that DM was triggered by Trastuzumab CD16-mediated antibody-dependent cellular cytotoxicity (ADCC) mechanism (10). Similar adverse reactions have been documented for Trastuzumab biosimilars (8). Trastuzumab Deruxtecan molecule consists of Trastuzumab, a cleavable linker and a topoisomerase I inhibitor. Studies confirmed that T-DXd retains Trastuzumab’s original functions after DXd conjugation: its Fab domain specifically binds HER-2 antigens on tumor cell surface, while the Fc domain engages Fcγ receptors on immune effector cells (e.g., NK cells), thereby resulting in antibody-dependent cellular cytotoxicity (ADCC) effect. The ADCC effect mediates tumor cell death through triggered release of cytotoxic granules (e.g., perforin and granzyme B) and cytokines (e.g., IFN-γ and TNF-α) (3). However, hypothetically, the ADCC effects of T-DXd may not be limited to tumor cells only and can potentially impact normal tissues. We speculate that T-DXd-induced tumor cell death may release large amounts of intracellular antigens, which could be recognized by the immune system as self-antigens, thereby resulting in autoimmune response. Simultaneously, activated immune cells (NK cells, T cells, macrophages) has ability to infiltrate muscle and skin tissues and release inflammatory cytokines (e.g., IL-6, TNF-α), leading to tissue damage. Further, immune system overactivation may lead to anti-ANA and anti-Ro52 antibody production closely associated with DM pathogenesis.

At the same time, paraneoplastic dermatomyositis (PDM) cannot be entirely excluded. This patient, aged over 40, developed dermatomyositis two years after she has been diagnosed with breast cancer. Skin biopsy revealed individual necrotic keratinocytes in the epidermis. According to the “International Guidelines for Cancer Screening in Idiopathic Inflammatory Myopathies” (26), this patient categorized as high-risk for developing IIM-associated malignancy. November 2024 and January 2025 tumor progression accompanied DM manifestation may explain PDM onset. Anti-TIF1-γ and anti-NXP2 antibodies are key markers of PDM, but, as limitation of this report, patient samples were not examined for these markers. Some studies demonstrated that DM patients have an elevated risk of tumor development, regardless of severity of DM effected muscles involvement severity (24, 27). Another studies demonstrated that cutaneous necrosis and an erythrocyte sedimentation rate (ESR) exceeding 40 mm/hour may be significant predictors of eventual malignancy development in DM patients (28). Although exact underline pathway remains unclear, it is widely believed that DM accompaniment of primary malignancy represents a paraneoplastic syndrome. Due to the tumor innate monoclonicity and ability to proliferate rapidly, malignancies often present autoantigens more frequently than typically encountered by the immune system, (including myositis-associated autoantigens). Non-specific muscle damage may cause myoblast proliferation, as a result, increased population of regenerating myocytes, which have been found to share antigens with myositis associated tumors (29). Combined with upregulated major histocompatibility complex (MHC)-I expression (30, 31), myocyte damage may be mediated by tumor-specific cytotoxic T lymphocytes (CTLs) that recognize shared antigens between regenerating myocytes and malignant cells (7). Although the direct pathogenicity of autoantibodies in DM remains unproven, myositis-associated malignancies may provide stimulus to potentiate host inflammatory responses.

It is also important to acknowledge the limitation of this study from the perspective of the medical oncology team with a key consideration, that there are three potential alternative therapeutic options for this case as well:

A) For such patient with an IHC result showing PD-L1 (CPS ≈ 30, clone SP263) and disease progression after treatment with Paclitaxel, Carboplatin, and Gemcitabine, alternative therapeutic options may include other chemotherapeutic agents combined with a PD-1 antibody, such as Pembrolizumab or Toripalimab.

B) This case patient’s diagnostic results indicated TROP-2 (3+). Therefore, Sacituzumab Govitcan or Sacituzumab Tirumotecan could also be an option.

The ASCENT trial (32) enrolled patients with advanced triple-negative breast cancer (TNBC) who had received at least two prior lines of chemotherapy. Participants were randomized to receive either Sacituzumab Govitecan or physician’s choice of single-agent chemotherapy (Capecitabine, Eribulin, Vinorelbine, or Gemcitabine). The study results demonstrated that in this group of previously received treatment patients with refractory TNBC, Sacituzumab Govitecan reduced the risk of disease progression or death by 59% and the risk of overall death by 52%.

Furthermore, OptiTROP-Breast01 (33) study evidenced that Sacituzumab Tirumotecan reduced the risk of disease progression or death by 69% compared with conventional chemotherapy. These results provide an effective treatment option for adult patients with unresectable locally advanced or metastatic triple-negative breast cancer who have received at least two prior lines of systemic therapy.

C) In case of germline BRCA1/2 mutation, treatment with Olaparib or Fuzuloparib in combination with Apatinib may also be considered.

In OlympiAD study (34), Olaparib significantly prolonged progression-free survival (PFS) of patients with germline BRCA1/2 mutated HER2-negative advanced breast cancer compared to chemotherapy (7.0 months vs. 4.2 months).

FABULOUS study (35) evaluated the PARP inhibitor Fluzuloparib, both as monotherapy and in combination with Apatinib, versus investigator’s choice of chemotherapy in patients with germline BRCA-mutated, HER2-negative breast cancer. As concluded, Fluzuloparib plus Apatinib combination significantly prolonged median progression-free survival (PFS) by 8.0 months compared to the chemotherapy group (11.0 months vs. 3.0 months; HR = 0.27, 95% CI 0.XX-0.XX).

This case describes the first report of a 43-year-old female with left breast invasive ductal carcinoma developed drug-induced dermatomyositis (DM) and interstitial lung disease (ILD) as rare seen unwanted drug reaction followed T-DXd therapy. Initially beneficial glucocorticoid therapy was not enough effective to prevent further DM symptoms aggravation after continued T-DXd use, that, to our conclusion, suggests cumulative effect of a drug, which may progressively worsened immunological complications. We speculated, that T-DXd triggered drug-related immune system dysregulation, affecting the lungs, skin, and/or muscles and leading to interstitial pneumonia and dermatomyositis. But the temporal overlap between breast cancer progression and dermatomyositis exacerbation without anti-TIF1-γ antibody testing does not allow us to definitively exclude possibility of paraneoplastic dermatomyositis (PDM). This findings address that T-DXd may trigger autoimmune responses through antibody-dependent cellular cytotoxicity (ADCC) or tumor antigen release, leading to multi-system involvement of the skin, muscles, and lungs. Clinicians must remain vigilant for such immune-related adverse events when using T-DXd, as early recognition and intervention are critical. In the era of immunotherapy, clinical application of T-DXd requires to rigorously consider possible immune-related adverse events, which demand early detection, and intervention to avoid irreversible and possibly fatal complications.

## Data Availability

The original contributions presented in the study are included in the article/**Supplementary Material**. Further inquiries can be directed to the corresponding author.
