# Supplementary material for: Case Report: Possible association between dermatomyositis and Trastuzumab Deruxtecan therapy of triple-negative breast cancer patient
Source: Front Immunol. 2025 Nov 10;16:1636581. doi: 10.3389/fimmu.2025.1636581 (PMC12640964; doi:10.3389/fimmu.2025.1636581)
Supplement: Supplementary file 1 [file DataSheet1.pdf]

CARE
case report guidelines

CARE Checklist of information to include when writing a case report

(cc) BY-NC-ND

Topic	Item	Checklist item description	Reported on Line
Title	1	The diagnosis or intervention of primary focus followed by the words "case report"	yes
Key Words	2	2 to 5 key words that identify diagnoses or interventions in this case report, including "case report"	15
Abstract (no references)	3a	Introduction: What is unique about this case and what does it add to the scientific literature?	18
	3b	Main symptoms and/or important clinical findings	27
	3c	The main diagnoses, therapeutic interventions, and outcomes	29
	3d	Conclusion—What is the main "take-away" lesson(s) from this case?	39
Introduction	4	One or two paragraphs summarizing why this case is unique (**may include references**)	44
Patient Information	5a	De-identified patient specific information.	67
	5b	Primary concerns and symptoms of the patient.	68
	5c	Medical, family, and psycho-social history including relevant genetic information	67
	5d	Relevant past interventions with outcomes	84
Clinical Findings	6	Describe significant physical examination (PE) and important clinical findings.	89
Timeline	7	Historical and current information from this episode of care organized as a timeline	127
Diagnostic Assessment	8a	Diagnostic testing (such as PE, laboratory testing, imaging, surveys).	95
	8b	Diagnostic challenges (such as access to testing, financial, or cultural)	101
	8c	Diagnosis (including other diagnoses considered)	107
	8d	Prognosis (such as staging in oncology) where applicable	123
Therapeutic Intervention	9a	Types of therapeutic intervention (such as pharmacologic, surgical, preventive, self-care)	110
	9b	Administration of therapeutic intervention (such as dosage, strength, duration)	112
	9c	Changes in therapeutic intervention (with rationale)	112
Follow-up and Outcomes	10a	Clinician and patient-assessed outcomes (if available)	
	10b	Important follow-up diagnostic and other test results	114
	10c	Intervention adherence and tolerability (How was this assessed?)	116
	10d	Adverse and unanticipated events	121
Discussion	11a	A scientific discussion of the strengths AND limitations associated with this case report	129
	11b	Discussion of the relevant medical literature **with references**.	129
	11c	The scientific rationale for any conclusions (including assessment of possible causes)	175
	11d	The primary "take-away" lessons of this case report (without references) in a one paragraph conclusion	247
Patient Perspective	12	The patient should share their perspective in one to two paragraphs on the treatment(s) they received	yes
Informed Consent	13	Did the patient give informed consent? Please provide if requested	Yes ☑ No ☐
